# Connected and confined: memories of digital experiences in relation to autonomy, competence, and relatedness during the COVID-19 pandemic

**DOI:** 10.1007/s12144-026-09895-9

**Published:** 2026-09-18

**Authors:** Joanna Witowska, Katarzyna Goncikowska, Christine Schoetensack, Vanda Černohorská

**Affiliations:** 1 Institute of Psychology, The Maria Grzegorzewska University, Szczęśliwicka 40, Warsaw, 02-353 Poland; 2 Institute of Psychology, Polish Academy of Sciences, Jaracza 1, Warsaw, 00-378 Poland; 3 School of Psychology, Liverpool John Moores University, Byrom Street, Liverpool, L3 3AF UK; 4 Institute of Philosophy of the Czech Academy of Sciences, Jilská 1, Prague 1, 110 00 Czechia

**Keywords:** Digital technology, Pandemic, Autonomy, Relatedness, Competence

## Abstract

The COVID-19 pandemic drove rapid digital transformation and disrupted the ways people could meet their basic psychological needs of autonomy, relatedness and competence. This study explored how people across six European countries (the UK, Poland, Czechia, Germany, Switzerland and Spain) recalled the role of digital technology (DT) in fulfilling these needs. 148 semi-structured interviews were conducted after the pandemic and were analysed using thematic analysis.

The analysis showed that DTs were recalled as both supporting and frustrating the satisfaction of all three psychological needs depending on broader material, institutional, social, personal and attitudinal conditions. Accordingly, DTs were associated with conditional autonomy and competence: In participants’ accounts, they were linked to experiences of both enhanced and diminished spatial and temporal empowerment and were tied to increased and reduced feelings of competence depending on factors such as time availability, living space, personal views of homeworking, organisational adaptation to digitalisation or external requirements relating to DT use. DT was also recalled in association with relatedness-enhancing experiences of social enrichment and relatedness-frustrating perceptions of disconnection and disengagement as a function of factors such as access to digital tools or cultural and social events, personal experiences such as emotional fatigue and digital norms.

These findings contribute to our understanding of how peri-pandemic digital experiences are reconstructed today and point to the persistence of memories that reflect context-dependent interpretations of how digital life shaped need satisfaction. Such memories suggest how psychological well-being during health crises and beyond may be jointly co-constructed by technology and society.

**Supplementary Information:**

The online version contains supplementary material available at https://doi.org/10.1007/s12144-026-09895-9.

## Introduction

Thanks to the Internet and emerging digital technologies (DTs), the beginning of the 21st century marked a transformative era of growth. The rapid adoption of smartphones, social media platforms, and cloud computing reshaped and expanded global connectivity (Ebert, 2011). Two decades later, the global COVID-19 pandemic changed the social, individual and economic aspects of life. It acted as a global catalyst, driving rapid digital transformation across multiple sectors (e.g. Vargo et al., 2021).

As Fuchs (p. 396, 2020) stated, “activities that humans usually perform in different social roles at different times in different locales converge in activities conducted in one universal, tendentially unzoned and unstructured space-time in one locale, the home”. The necessity of pursuing multiple tasks such as work, social, family and free time activities, at home and sometimes simultaneously, posed a threat to the fulfilment of core psychological needs including autonomy, competence and relatedness (Deci & Ryan, 2000). At that point, DTs likely contributed to individual resilience or the ability to remain relatively unaffected by such challenges as they not only supported, but also enabled everyday tasks, e.g. communication, work, education, and social interactions (Papagiannidis et al., 2020; Starkey et al., 2021), which were essential for the fulfilment of needs.

Deci & Ryan’s (2000) model of basic psychological needs (BPN) identifies three core needs: autonomy, competence and relatedness. Autonomy refers to the feeling of having choice and willingly endorsing one’s actions, as opposed to feeling compelled or controlled. Competence reflects the experience of mastery and effectiveness in one’s activities, fostering a sense of accomplishment. Relatedness encompasses the need to feel connected to others and experience a sense of belonging and meaningful relationships.

The role of DTs in the fulfilment of BPN has attracted considerable attention among researchers (e.g. Brown & Greenfield, 2021; Costa et al., 2022; Courbet et al., 2023) as meeting such needs has been found to be critical for well-being (Church et al., 2013; Deci & Ryan, 2000). It seems that the role of DT in BPN satisfaction is highly contextual and complex. For example, digital education can enhance autonomy through the provision of flexible choices regarding time and place of study, self-paced modules or personalised learning options, whereas relatedness needs may be more difficult to meet in online learning environments as these may hinder meaningful interaction with peers and teachers (Salikhova et al., 2020). On the other hand, DT can support relatedness by fostering social empowerment and social connection among older adults (Hill et al., 2015). However, a study conducted among students in Pakistan showed that increased use of social networking sites was associated with greater need frustration and in turn led to lower satisfaction of all three BPN (Mahrukh et al., 2024). Moreover, affordances theory states that affordances of specific platforms or devices may shape BPN. For instance, a study conducted among TikTok users revealed that hedonic affordances of TikTok were positively associated with autonomy satisfaction; connective affordances were correlated with relatedness; and utilitarian affordances positively correlated with competence satisfaction (Song et al., 2021).

Previous studies conducted during the pandemic demonstrated that the pandemic created barriers to meeting needs (Avsec et al., 2021; Cantarero et al., 2020). External regulations restricted individual freedom, reduced opportunities for social connection and in general limited the choice over how, where, when and what everyday activities could be pursued, thereby impacting well-being.

Remote work and its impact on autonomy were the subject of extensive research during the pandemic. For some, remote work offered flexibility, comfort, and time savings by eliminating commutes, allowing for greater control over tasks and personal activities (Fukumura et al., 2021; Mullins et al., 2022). However, this autonomy was undermined by pressure to remain constantly available, which contibuted to work intensification due to excessive online meetings or the failure to adapt workloads to home working arrangements (Abgeller et al., 2024; Fiorini, 2024; Kunze et al., 2020; Oakman et al., 2022; Psalti & Antoniou, 2023). In education, the rise of remote learning appeared to strengthen learners’ sense of autonomy when they were able to meet the self-management demands associated with online study, such as planning their learning and selecting their own learning methods (Achuthan, 2025; Chiu, 2023). The ability to engage effectively in self-regulated and self-directed learning was likely influenced by contextual factors, such as the availability of digital support (Chiu, 2022), e.g. multimodal learning resources (Hoi, 2022) or learning strategies (Irgatoğlu et al., 2022), which have been associated with increased autonomy. In contrast, insufficient instructor support, limited adaptation of teaching methods to the online environment and pressure to conform to such methods tended to undermine learners’ perceived autonomy (Hoi, 2022).

Beyond work, the ability to make choices during free time was constrained by external regulations as well. Reduced non-digital options appeared to contribute to greater reliance on digital entertainment. Some individuals described a reduced sense of autonomy because of hard-to-limit unrestricted and repetitive content (Ganter, 2025). The rapid digital transformation required employees to adopt new skills, digital competencies and behaviours in order to thrive in remote work. Some individuals experienced difficulties in this process that could affect their success at work depending on situational factors, such as organisational support or resources (Henke et al., 2022). Many employees lacked essential skills so that these had to be acquired, which was recalled as a source of stress that participants associated with reduced productivity or job satisfaction (Bhattarai, 2020; De’ et al., 2020; Schmitt et al., 2021; Toscano & Zappalà, 2020). The educational sector faced similar transformations and challenges. Online education increased accessibility and flexibility while at the same time causing psychological stress and difficulties adapting to virtual platforms (Dhawan, 2020; Hossain et al., 2021; Yu-Fong Chang et al., 2021), as well as exposing barriers to accessing and using digital services (Blomberg et al., 2021; Ragnedda et al., 2022; Zheng & Walsham, 2021). As digital demands increased, technology-related self-efficacy declined among both college (Alemany-Arrebola et al., 2020; Hilpert et al., 2022).

Research on the role of DTs in maintaining meaningful social relationships demonstrates mixed findings. DTs facilitated social connection with family and friends, helped fulfil social needs, and reduced anxiety under conditions of isolation, underscoring their importance when in-person contact was limited (Shah et al., 2020; Stuart et al., 2021). However, excessive use was linked to negative well-being outcomes (e.g. Popescu et al., 2022) and online relations often lacked the depth of face-to-face interaction. Face-to-face interaction is widely considered the most basic and fundamental way humans connect and communicate with each other. According to social presence theory, digital media differ in their capacity to facilitate social contact (Oh et al., 2018). In-person interactions are rated as highest in social presence, while computer-mediated communication (video/phone calls) facilitates social presence only to some extent (Fox & McEwan, 2017). Hence, some of the studies show that online forms of social interaction during the pandemic could not compensate for face-to-face contact (Rouxel & Chandola, 2023).

## The current study

As the reviewed research suggests, a substantial number of studies provide insight into how DT may have been related to basic need satisfaction during the pandemic, painting a comprehensive picture of peri-pandemic digital experiences. However, it is currently unclear what aspects of peri-pandemic digital life and its role in need satisfaction remain salient in individuals’ recollections of the COVID-19 period today. Hence, in the current study, we were interested in what retrospective accounts of pandemic digital practices reveal about the relationship between DT and psychological needs.

The limits as well as the value of exploring retrospective narratives of digitally mediated need satisfaction lie in the reconstructive nature of memories. Remembering involves a reconstruction of past experiences (Fivush & Grysman, 2023) that is shaped by various factors, such as psychological priorities (Halamish & Stern, 2022), knowledge (Migueles & García-Bajos, 2012) and cultural context (Wang & Ross, 2005). Consequently, retrospective accounts tend to blend actual experiences with inferences and interpretations and may not faithfully reflect how events were originally perceived.

However, such narratives are valuable for understanding how people currently make sense of the past. Capturing this is important since the meanings attributed to experiences can shape future behaviour and attitudes (Kuwabara & Pillemer, 2010). Retrospective narratives of experiences can thus be an important source of data to predict likely digital attitudes in future crises, which in turn may support more targeted and effective preparation of public health responses to such crises.

Investigating memories of DT use also allows for the identification of digital experiences that were particularly novel, meaningful or emotionally impactful as salient experiences are more readily encoded and recalled (Neisser, 1986; Hjuler et al., 2025). Shared recollections across individuals may signal collective significance, highlighting areas requiring attention in future digital well-being strategies.

In addition, unlike studies conducted during the pandemic, the investigation of past digital experiences offers the opportunity to analyse narratives of processed experiences, which are less likely to be influenced by strong emotions and feelings that might make autobiographical memories more general, less vivid or difficult to remember (Rohde et al., 2024; Klier & Buratto, 2020).

Given the benefits and implications of analysing how digital life during the pandemic is remembered today while also considering the limitations of retrospective accounts, our study drew on interview data collected in six European countries. We explored participants’ recollections of the ways and contexts in which DTs satisfied and frustrated their needs for autonomy, competence and relatedness during the pandemic. While the interviews conducted focused on how DT was used in everyday life without enquiring specifically about digital experiences during the pandemic, peri-pandemic DT use was addressed spontaneously by participants.

## Method

### Participants and procedure

This study’s methodology is based on the exploratory phase of the TIMED project, which aimed to identify key themes related to digitalisation and time perception. As part of the data collection linked to the TIMED project, 300 interviews were conducted: six teams from the Czech Republic, Germany, Poland, Spain, Switzerland, and the UK each conducted 50 interviews in their respective countries. Data collection took place between December 2022 and May 2023, using face-to-face, telephone, and online interviews. The average interview duration was 51 min. All participants provided recorded informed consent, either verbally or in writing, and all the interviews were audio-recorded. Ethical approval was obtained in each participating country, and the study was conducted in accordance with the ethical standards as laid down in the 1964 Declaration of Helsinki. To capture diverse experiences and perspectives, the interviews included individuals from a variety of backgrounds. These backgrounds were categorised into specific groups ensuring a broad cross-section of views. The strategy targeted individuals with different levels of immersion in digital life and from various employment sectors. We also included students and retired as well as unemployed individuals. This recruitment strategy was based on stakeholder input and literature showing that DT use and time experience vary by gender, age, socioeconomic status, education, and occupation (Bela et al., 2020; Elena-Bucea et al., 2020; Twenge & Martin, 2020).

Of the full set of 300 interviews, 148 participants explicitly reflected on the pandemic. 152 interviews did not contain unprompted references to the pandemic and were therefore excluded from the analysis. 148 interviews were analysed in the present paper. The corresponding 148 participants included both women and men (56% and 44%, respectively), aged 18–91 years (*M* = 42.0, *SD* = 16.1), with women (*M* = 43.0, *SD* = 16.4) and men (*M* = 41.0, *SD* = 15.7) showing comparable age distributions. Participants were residents of Switzerland (22.3%), the United Kingdom (21.6%), Czechia (18.9%), Poland (17.6%), Germany (10.8%), and Spain (8.8%). With respect to education, most participants held a bachelor’s degree or higher (68.2%). Among women, 24.4% reported completing secondary education and 4.9% vocational education, while among men 20.3% had completed secondary education and 10.9% vocational education. Employment status was diverse. Most participants reported being in gainful employment (“any occupation”: 70.0%), while 14.2% were university students and 12.5% inactive in the labour market (e.g., retired, unemployed, or engaged in caregiving). Only one participant (0.8%) reported “other” occupational status. For details on participant demographic characteristics, specifically gender, age and education by country, see Tables 1a and 1b in the supplementary material.

### Analytical strategy

The data was analysed using inductive, data-driven, and interpretative thematic analysis (Braun & Clarke, 2006). Each research team used NVivo or MAXQDA to examine their data through an iterative coding process that began during data collection. Initial codes were generated inductively from participants’ accounts, focusing on recurring meanings, experiences, and patterns identified within the interviews. Weekly team meetings helped develop a shared codebook, which included definitions and illustrative quotes. The codebook was continuously updated through the addition of new codes and refinement of existing ones to capture emerging themes. During these discussions, related codes were compared and grouped into broader candidate themes, allowing researchers to identify patterns both within and across datasets. Throughout the analysis, the authors upheld reflexivity by engaging in introspection and mutual discussions about their individual coding processes and decisions. This collaborative effort aimed to enhance the credibility of the analysis and its interpretations (Yardley, 2000). To ensure consistency, 10% of interviews were double-coded by at least two researchers. Some of the analysed data has been published by TIMED, however, the publication(s) do not overlap with the analyses presented in this paper.

In the present study, we focused on pandemic-related experiences. Interviews were selected for analysis if participants made explicit and unprompted references to COVID-19 and its consequences. Full transcripts were reviewed, while pandemic-related extracts were systematically identified using the consortium’s structured data harvesting template. To identify the relevant material, the consortium co-developed a structured data harvesting template specifically for the pandemic-focused analyses. The harvesting process was guided by the preliminary research question: “What do retrospective accounts of peri-pandemic digital practices reveal about the relationship between DT and psychological needs?”, which informed both the selection of relevant interview excerpts and the development of thematic categories. This framework was designed to ensure consistency in extracting relevant material from the national interview datasets while allowing flexibility for country-specific contexts. Each team completed this template for their interview set and the combined material was used to extract themes across countries. The template included preliminary thematic areas to be considered during data extraction, accompanied by short descriptions and guiding questions, as well as space for illustrative quotations in both the original language and English. Each team completed the template for its interview set, and the consolidated material was used to identify and refine themes across countries. The final thematic structure was reviewed iteratively through consortium-wide discussions, during which thematic boundaries, interpretations, and cross-country consistencies were examined and refined collaboratively.

## Results

Overall, DTs were remembered as both facilitating and hindering autonomy, competence and relatedness during the pandemic. Crucially, however, need satisfaction was perceived to have been shaped not only by DTs themselves but by their interaction with separate need enabling and frustrating conditions. An overview of such conditions across all three need domains is provided in Table 2. The table also connects these conditions to four main themes, which were established to capture key participant memories of digital experiences relevant to autonomy, competence and relatedness: (1) Digitalisation contributes to conditional autonomy, (2) Digitalisation contributes to conditional competence, (3) Digital social enrichment and (4) Limitations of digital relatedness. The following paragraphs describe each theme along with corresponding subthemes.

**Table 2:** Enabling and frustrating conditions for autonomy, competence, and relatedness in retrospective accounts of digital technology use during the COVID-19 pandemic

Need	Theme	Conditions supporting need satisfaction	Conditions frustrating need satisfaction	Illustrative participant contexts
Autonomy	Temporal empowerment	Time abundance (furlough, reduced obligations); flexible or self-set work schedules; availability of digital leisure and entertainment options	—	Exercise and fitness apps used during lockdown (UK); flexible part-time remote work allowing self-planned schedules (PL)
	Temporal disempowerment	—	Caregiving responsibilities colliding with remote work; work–life boundary collapse; involuntary multitasking; organisational overload and unchanged workload expectations	Mothers completing work at night due to childcare during the day (CZ); expectation of five Zoom meetings per day becoming the new performance standard (CZ)
	Spatial empowerment	Access to private and undisturbed home space; availability of personal digital devices and a dedicated workstation; perception of remote work as a sign of organisational trust	—	Spacious apartment enabling quiet, separated work (CH); home digital equipment interpreted as employer trust and independence (CZ)
	Spatial disempowerment	—	Shared or overcrowded living space; shared devices; inability to maintain privacy; no physical separation of work, family, and self-care domains	Psychotherapy clients unable to speak freely due to risk of being overheard at home (ES); shared computer preventing access to digital healthcare services (CZ)
Competence	Struggling to keep pace with digital demands	—	Flawed or delayed provision of digital infrastructure by public or governmental bodies coupled with lack of user support; unfamiliar or faulty digital processes; unsuitability of digital tools for embodied, practice-based or sustained-concentration learning; prolonged video sessions exceeding concentration limits; digital fatigue and distractibility; new digital demands of children’s education exceeding parental digital skills	QR-code-only employment service with no alternative access and no in-person staff (ES); parent overwhelmed by Teams used for child’s schoolwork (e.g. PL/UK accounts); four-hour video lessons perceived as exceeding sustainable concentration (CH); students unable to explain ideas in group work without face-to-face contact (CH)
	Growing through digital accomplishments	Organisational adaptability and creative problem-solving; alignment of digital solutions with personal and professional values; successful team collaboration via digital tools; availability of remote work (instead of furlough); support from employers and team members	—	Remote camera operation enabling continued film production during lockdown (UK); large-scale remote training of customers on COVID-19 online course; digital training rollouts allowing rapid upskilling of large numbers of employees
Relatedness	Digital preservation of social life	Access to video-conferencing platforms; geographic separation making digital contact the only available means of connection; institutional provision of devices (e.g., hospital tablets)	—	Zoom used for birthdays and seasonal celebrations (UK, DE); hospital tablets enabling patients to maintain contact with loved ones (CZ); WhatsApp video calls with parents initiated for the first time during lockdown (DE)
	Digital participation	Pandemic-driven openness to new platforms; formation of new online interest-based communities and group chats; access to live-streamed cultural and social events	—	New Facebook community groups and university friend chats formed during the pandemic (UK, PL); virtual live concerts with real-time audience interaction experienced as meaningful social participation (UK)
	Digital disconnection	—	Absence of physical presence, touch, and spontaneity; emotional fatigue from sustained screen-based interaction; perceived superficiality of digital exchanges compared to in-person contact	Participants consciously limiting screen time due to emotional drain (DE); friendships described as “losing their way” due to exclusively digital contact during lockdown (UK)
	Digital disengagement	—	Camera-off norms in online meetings; no informal contact opportunities (e.g., corridor, kitchen); new working relationships formed exclusively online perceived as lacking depth and cohesion	New colleagues never seen on camera during onboarding (CZ, PL); loss of casual encounters and small talk in hybrid and remote working arrangements (UK)

Note. Conditions were identified through inductive thematic analysis of retrospective interview accounts (Braun & Clarke, 2006). Need domains follow Deci and Ryan’s (2000) Self-Determination Theory. Country codes: *CH =* Switzerland; *CZ =* Czechia, *DE =* Germany; *ES =* Spain; *PL =* Poland; *UK =* United Kingdom. Em dashes (—) indicate that no conditions of that type were identified for the given theme. Theme numbering corresponds to the Results section

### Autonomy: Digitalisation contributes to conditional autonomy

Participants’ memories suggested that their experience of autonomy was associated with the interaction of DTs with factors such as participants’ general time availability (how much time they had available in life), pre-existing access to undisturbed living space and digital equipment, as well as the way they appraised confinement to the home in their specific cultural context. In other words, DTs were recalled as enhancing self-determination only under certain circumstances and were therefore perceived as contributing to conditional autonomy. We identified two main themes which describe how digitalisation was perceived to have influenced Temporal (dis)empowerment and Spatial (dis)empowerment in interaction with socially determined factors.

#### Temporal (dis)empowerment

Some participants recalled that confinement to the home during the pandemic and restrictions imposed upon face-to-face socialising contributed to a reduction in activities that they could fill their time with. Inability to do paid work due to pandemic-related furlough, limited outdoor activities and social free time pursuits created an excess of time that had to be managed. Under these circumstances, digital entertainment, such as the consumption of social media content and digitally supported leisure, e.g. walking or exercising accompanied by the use of apps, was reported to either assist participants to simply eliminate boredom or enrich their free time with meaningful leisure. For example, DTs were described as facilitating physical exercise at home, thereby supporting the maintenance of healthy routines. They were also perceived to enable the comforting remote “connection” with culturally relevant figures of public interest or content and, according to participants, even led them to initiate and enjoy the use of digital entertainment services, such as TikTok, which they had previously not been familiar with. Under conditions of time availability, DTs were therefore reported to allow participants to actively fill and shape their free time meaningfully, with DTs providing a range of activities to choose from.

*They’ve got like hundreds of different [exercise] videos. So they do body pump*,* you know everything*,* but you can choose on their app*,* you pay for it for a year. But we found it absolutely vital in COVID to carry on*,* you know*,* exercising and then not just sit there drinking like*,* like most of us. Uh*,* so yeah*,* we do that. I think the [exercise app] is really important to us. (Probation officer*,* F*,* 45*,* UK)*

*Of course*,* during the pandemic*,* I also tuned into various things of my ‘favourites’*,* in quotes*,* celebrities*,* because it’s such an ugly word*,* about these famous people who also somehow tried to make this difficult time easier for themselves and others. So*,* in those moments*,* I definitely make the most of such things. From various instructional videos*,* precisely. (Babysitter*,* F*,* 52*,* Poland)*

For some participants, remote work allowed for temporal autonomy, offering flexibility in choosing when to work rather than adhering to fixed office hours. However, this positive view was more common among those with greater time abundance. One part-time worker noted that while DT was essential for staying employed, it was the freedom to set one’s schedule, not just remote work itself, that fostered temporal autonomy.

*It wasn’t a full-time job, so I think it could have been up to 4–5 h. It wouldn’t be possible without these technologies. And generally, I didn’t feel much of a difference. I also didn’t have fixed working hours, so I worked when I wanted, so I didn’t feel much negative influence. There was nothing like spending half a day in pyjamas, I just planned it myself.* *(Simulated patient at medical school, internship as UX designer, F, 21, Poland)*

For some time-poor participants with caregiving responsibilities, the flexibility of teleworking was not always recalled as leading to the desired temporal autonomy. Remote work was often described as blurring the lines between work and family time, as both had to be managed simultaneously. Alternatively, if separated, work could extend into the evening due to daytime interruptions. In this regard, one Czech participant highlighted how the individualistic concept of autonomy promoted the unrealistic idea that work-life balance could be achieved by women with children through effort, noting the negative implications of home working on primary carers’ (mostly women) energy and concentration during the pandemic.

*That naive idea that you just have to try a little bit more and lean in […] I am convinced that working from home with a little child who is either not in kindergarten or is home sick is hell. You have to focus on your child anyway*,* which makes it hard to concentrate. So*,* you end up doing the work at night*,* right*,* because you’re taking care of the child during the day. […] It’s extremely mentally exhausting and*,* of course*,* physically exhausting. (Union expert*,* F*,* 42*,* Czechia)*

#### Spatial (dis)empowerment

Several participants alluded to the role of living space in shaping the sense of self-determination that DTs could afford during the pandemic. For example, remote work was portrayed as “easy” and comfortable when shared living arrangements still allowed for spatial separation from other individuals, such as family members, so that a sense of privacy and an undisturbed environment could be maintained. In addition, the ability to set up one’s own workstation at home with sufficient digital equipment was also reported to increase the ease with which different work tasks could be organised, separated and integrated with leisure. Conversely, when interpersonal proximity at home could not be avoided, the digitalisation of services, such as healthcare, became an obstacle. This can be illustrated by the quote of a psychotherapist who reflected on clients’ difficulties in speaking freely due to the risk of being overheard in their own home. Access to private space equipped with suitable digital devices partly determined by socioeconomic factors, therefore, seemed to shape the extent to which DTs could contribute to keeping remote work processes, free time and self-care under control.

*Well*,* it was easy in the sense that maybe I’m lucky enough to have an apartment where there’s a bedroom for me and one for my husband’s*,* and my two daughters have one too. […] So we weren’t on top of each other*,* and we could work quietly and then get together for a break*,* so it was pretty easy. (Service assurance*,* F*,* 50*,* Switzerland)*

*For example*,* as a psychologist*,* I felt very limited in my work. It’s very different from meeting in a space completely outside their homes […]. Plus*,* when there were complex home situations*,* like sharing one computer*,* it made things more difficult. Family issues also came up*,* like […] ‘I can’t talk because my dad will hear me.’ That was difficult. (Psychologist, currently not practicing*,* 30*,* Spain)*

However, according to participant accounts, remote work was influenced not only by external factors like space and equipment but also by how confinement to home during working hours was perceived. Some people viewed the “obligation” to work remotely as an empowerment and comfortable arrangement rather than a restriction. The pandemic was seen as having forced employers to adapt work modes to employees’ needs, reducing commutes and increasing organisational openness. One participant even saw the use of home digital equipment as a sign of trust, suggesting that home working represented greater independence for employees.

*[Bank XY] is a pretty rigid company regarding home offices or any remote work. And with COVID*,* that changed a lot because almost 90% of the people stayed at home for various reasons*,* and suddenly*,* we just had to learn to work remotely. Initially*,* it was a massive benefit for those people; just because I could sit at home*,* I didn’t have to go to the office*,* which was nice. (Teacher*,* F*,* 33*,* Czechia)*

In contrast, a few other participants portrayed working from home during the pandemic as a way for their organisations to reap financial benefits, e.g. by reducing operating costs of separate offices which were unused when home working was implemented or by taking measures to increase “productivity” of employees at their cost. Such measures included failing to readjust expectations regarding employees’ work pace during remote work, e.g. digital meetings were replaced by hybrid working, the latter of which was perceived to require a slower work pace. In other words, some participants viewed home working as a tool of power used by organisations to control employees’ workplace, work tasks or pace, which was associated with a reduction in their personal autonomy. In line with this, other interviewees reported a simple conflict between their desire for non-digital modes of working and studying and the implemented teleworking and online studies.

*[After some time*,*] people didn’t want to work from home anymore. The employees were fed up; they wanted to go back to the office. Companies took advantage of the pandemic and started to see it as an opportunity to reduce operating costs. When people are at home*,* employers save on offices*,* heating*,* and work tools*,* and I don’t know what else. (Union expert*,* F*,* 42*,* Czechia)*

*My experience during COVID*, * for example*,* was that I could get much more work done because I eliminated the commute. On the one hand*,* it is great when you’re dealing with childcare and taking care of little ones because you’re glad for every extra minute. On the other hand*,* it leads to taking on an abnormal amount of work*,* like some overload. It also leads to social isolation. And then it’s difficult for the people and their employers to say there are two performance standards suddenly. You get used to that person on Zoom doing five meetings a day. And then suddenly*,* when you’re not entirely online anymore*,* and in hybrid or completely offline mode*,* you can’t expect people to do five meetings a day anymore. (Union expert and lawyer*,* F*,* 42*,* Czechia)*

### Competence: Digitalisation contributes to conditional competence

While reflecting on the pandemic, participants shared memories of both a reduced sense of competence but also enhanced self-efficacy associated with their use of DT. Exposure to unfamiliar or flawed digital processes seemed to result in feelings of frustration, incompetence, or being overwhelmed. In addition, some participants reported facing cognitive, didactic and interpersonal challenges when using DTs in work and education, which interfered with concentration, teaching and communication. Conversely, others remembered an enhanced sense of accomplishment associated with the digitalisation of work life when digitalisation was perceived to facilitate work efficiency, innovation or collaboration.

Two themes were established to capture these diverging participant experiences of competence: Struggling to keep pace with digital demands and Growing through digital accomplishments. As in the case of autonomy, the extent to which digitalisation was remembered as enhancing perceived competence depended on contextual factors. These included organisational adaptation to digitalisation, the ways participants were required to use digital tools in work and education and the availability of remote work and support from employers.

#### Struggling to keep pace with digital demands

The retrospective narratives of participants suggested that digitalisation could contribute to feelings of incompetence or frustration as participants struggled to use digital tools to effectively meet their own or their families’ essential needs, such as ensuring their own financial stability or supporting their children’s education. For example, parental difficulties in helping children navigate digital software, such as Teams for schoolwork could result in parents feeling overwhelmed as they struggled to keep up with the new digital demands of their offspring’s education. Other participants faced significant frustration when they found themselves unable to use digital processes, such as accessing financial aid remotely. However, rather than attributing such experiences of competence frustration merely to digitalisation itself or their own shortcomings participants suggested that it was the flawed or delayed provision of digital infrastructure by public or governmental organisations coupled with a lack of user support that contributed to such competence frustration. For instance, one service user reported having been instructed to use a faulty QR code by an employment support service to claim benefits, which ultimately did not allow them to access financial aid and left them feeling frustrated and incompetent due to a lack of effective digital infrastructure in their country.

*During the pandemic*,* we had the problem that I could not connect with SEPE (State Public Employment Service)*,* but it was not a problem of mine*,* it was a general problem in Spain. I went to the SEPE in person*,* you couldn’t get in*,* there were no people and they showed me a QR code and to do it through a QR code. […] The lady who was telling me with this QR*,* you will get to a green paper. […] Well*,* from January they took away my financial aid. (Retired accountant*,* F*,* 60*,* Spain)*

After digital service provision and operations had become more established within public bodies, difficulties keeping up with digital demands and retaining usual levels of perceived competence in work and education seemed to emerge among employees and students. They attributed such competence frustration to a perceived unsuitability of digital tools or the way they were employed for work or learning processes that heavily benefitted from face-to-face interpersonal contact or embodied learning or required sustained concentration. For example, one educator described difficulties teaching practice-based social work skills remotely because the digital medium she was expected to use for didactic purposes, i.e. videocalls could not replicate the experiential aspects of learning. In line with this, university students and employees reported problems concentrating or communicating, digital fatigue and distractibility during online meetings or lectures, which was associated with reduced feelings of achievement and poorer educational performance as the way DT was employed in teaching, e.g. in the form of extended online classes was considered to interfere with focus, hamper group communication or allow for simultaneous engagement in other distracting activities. Thus, according to retrospective accounts, competence frustration seemed to occur when digital demands exceeded participants’ existing personal, social or cognitive capabilities.

*If you have 2 hrs of video lessons, for me, it doesn’t bother me at all, I like it. But when you have 4 hrs of video lessons, it’s too much and you can’t maintain concentration for 4 h, it’s not possible. Well, I don’t know if some people manage it, but I don’t think it’s possible. (Student, F, 21, Switzerland)*

#### Growing through digital accomplishments

Several participants recalled enhanced competence at work specifically when new digital tasks or work arrangements allowed them to work more efficiently, innovate or collaborate more effectively than before the pandemic. For example, some participants recalled increased work productivity due to the removal of commuting time made possible by remote work. Another individual shared memories of enhanced work efficiency as she trained many customers remotely through a COVID-19 online course. Similarly, the collaborative development and implementation of innovative digital solutions, such as the remote operation of a camera on a film set were remembered as significant accomplishments. While reflecting on such digital achievements, participants communicated an awareness that contextual factors such as the availability of remote work (instead of furlough) and support from employers or team members contributed to their experiences of growth and competence.

*My job is essentially 30 people climbing on top of each other in a small space. That’s what film sets are. […] With COVID*,* that couldn’t happen. But the industry has kind of bent over backwards to find ways around that to make it work. So quite often they’ll use a*,* instead of me physically holding the camera and moving it they will put it on a remote head so I can be in a different room with controlling the movement. […] there is no group of people in the world better at solving problems than a film crew. So they*,* it was incredible the hoops we jumped through to get back to work. […] It was brilliant. (Camera operator*,* M*,* 46*,* UK)*

### Relatedness: Digital social enrichment

For many participants, DTs were a vital means of maintaining social bonds during the pandemic, as they helped individuals to fulfil the need of relatedness under restrictive conditions. In the absence of face-to-face contact, digital tools allowed people to continue interacting with friends, family and professional networks. In some cases, these forms of mediated communication preserved existing relationships and fostered new connections and social participation. To describe such forms of social enrichment, two key themes were identified: Digital preservation of social life and Digital participation.

#### Digital preservation of social life

During the pandemic, many individuals became heavily reliant on DT. Platforms like social media and video conferencing tools such as Zoom became vital substitutes for in-person interactions, helping to mitigate isolation and maintain relationships. Zoom, in particular, became central to sustaining distant social networks, emphasizing its perceived long-term significance beyond the lockdown period. DT was referred to as “a lifeline”, highlighting how, in the absence of physical gatherings, it became the primary means of connection, accessible “all day, every day”. Celebratory events, such as birthdays, and seasonal festivities, along with regular daily interactions, were facilitated through virtual meetings, thereby establishing novel patterns of interaction. In environments like hospitals, digital tools played a transformative role, with tablets allowing patients to stay in touch with their loved ones. These digital solutions provided a sense of continuity and belonging during a period of social disruption.

*I think during COVID it [digital technology] was probably a lifeline. Because it was the only way you could communicate*,* wasn’t it*,* with people*,* because you couldn’t go and see them*,* unless you were Boris Johnson. (Laughter). So yes*,* during the pandemic it was an absolute lifeline. And it was available to use all day*,* every day*,* because there was nothing else to do. (Admin role*,* F*,* 65*,* UK)*

*Very quickly*,* the hospitals bought tablets; through those tablets*,* the patients could have contact with their loved ones. It was great that*,* suddenly*,* technology could make life more comfortable again in a way that we wouldn’t have dared to do. (Head of university department and psychologist*,* M*,* 42*,* CZ)*

*Wasn’t it during the Corona period? That’s when video telephony really started in private life. Yes*,* before that it was kind of an exception to talk to each other on Skype. It was only done when a good friend was in Australia. Yes*,* then we used to Skype. Exactly that*,* talking to each other via WhatsApp video calls. Yes*,* in the corona times*,* when you had to be home by 19:00*,* I even started video calling my parents*,* because otherwise you couldn’t see each other at all. (Team leader*,* F*,* 40*,* Germany)*

#### Digital Participation

Digital platforms allowed for the emergence of new forms of social participation. One interviewee observed that the pandemic was associated with a greater awareness of the potential of technology, noting the creation of group chats that had not existed previously. Some participants engaged in reconnecting with former colleagues via a Facebook group, while others established a university friends chat that didn’t exist before the pandemic. By joining an online Facebook community one participant was able to engage with others and find companionship, which demonstrates how online spaces could provide meaningful social support *(Babysitter*,* F*,* 52*,* PL)*, enabling individuals to cultivate relationships and maintain a sense of belonging despite physical isolation.

Online events, such as live concerts, instructional videos, and virtual meetups, became key ways to engage socially and culturally. Some participants mentioned turning to online content from public figures and instructional materials for comfort, engagement and as a source of inspiration. Others described engaging with live-streamed concerts and interest-based communities on platforms such as Facebook, where they could interact through chats and discussions. For example, some enjoyed late-night virtual concerts hosted by their favourite music groups, while others participated in niche interest shows, such as metal detecting, which allowed for real-time interaction and community building*.* That participant described the experience of watching virtual concerts with online chats as “amazing”, noting that it enabled her to interact in meaningful ways with others despite the physical isolation.

*“[The pandemic] opened our eyes a bit more to like what you can do with technology” (Mental health support worker, F, 26, UK)*

*When I’ve been on Facebook […]*,* especially during COVID*,* there were a lot of groups that*,* music groups that I like that were doing concerts*,* you know*,* like in their homes and stuff*,* and it was broadcast. It was in the middle of the night at 1:00 o’clock in the morning cause most of them were in America. But that was amazing to interact that way*,* obviously that you have a chat that you can type in*,* you know*,* sometimes you get a discussion back or sometimes I’ll watch*,* I’m into metal. (Kitchen porter*,* F*,* 58*,* UK)*

### Limitations of digital relatedness

Despite being remembered as facilitating continued social interaction during the pandemic, DTs were often recalled as falling short of fulfilling individuals’ deeper need for emotional connection and belonging. Participants conveyed how digitally mediated contact lacked the intimacy, spontaneity, and embodied presence characteristic of in-person relationships. While technology provided a functional means of communication, many narratives pointed to a relational void characterised by emotional fatigue, superficial exchanges and a sense of social alienation that describes a “societal loss”. Two central themes were established to describe this phenomenon: Digital disconnection and Digital disengagement*.*

#### Digital disconnection

Despite the benefits of DTs, there was an undercurrent of ambivalence. For many, online connections fostered “a kind of closeness”, but this virtual closeness was insufficient to replace the depth and richness of face-to-face interactions. This duality reflects the complex nature of “digital socialization” – valuable in maintaining contact, yet insufficient for fulfilling the deeper emotional and relational needs typically met through in-person encounters. Despite frequent virtual engagement, the lack of physical presence was accompanied by feelings of disconnection. Some participants emphasised a preference for traditional, face-to-face interactions instead of online substitutes:

*Moreover*,* during the pandemic and all of this*,* a person… wants to*,* at least for me… have contact with people*,* not machines. (Pensioner – office worker/secretary*,* F*,* 51*,* Poland)*

*During lockdown again*,* there was the odd Teams calls or the Zoom quizzes*,* there was a lot of that sort of stuff going on. Umm*,* but again […] I’m not old school with it*,* but I just think if I can go and meet someone*,* I’d probably rather do that than pick up the phone […]*,* interacting with somebody face-to-face is always good. (Underwriting training specialist*,* M*,* 47*,* UK)*

Over-reliance on digital communication left some participants feeling emotionally drained, leading to conscious efforts to limit screen time. Some felt that digital communication had not only failed to bridge the divide but had appeared to contribute to feelings of erosion of social ties. These experiences suggest that while digital tools were recalled as having been essential during the pandemic, they also highlighted what was missing – spontaneity and the nuanced dynamics of physical presence.

*For me*,* it was a depressing change*,* sitting in front of the computer alone. (Management*,* NGO project coordinator*,* F*,* 44*,* Poland)*

*I’ve also used Telegram and WhatsApp before*,* to the same extent before and of course more during the pandemic. So*,* I mean*,* if it’s the only way to stay in touch. But as I said*,* I couldn’t say that I’ve become more digital in my private life. I try to avoid it. Yes*,* because I simply realise that it’s not good for me. It tires me out. I just want to have other options. (Cook*,* F*,* 60*,* Germany)*

*During COVID*,* everything obviously was digital*,* wasn’t it […] I noticed like a lot of erm*,* yeah*,* I think a lot of like friendships almost like lost their way a little bit because it was only digitally as opposed to meeting up in person*,* it wasn’t as easy to like do that*,* it wasn’t as easy to catch up with people […] so I*, *from that point of view*,* it kind of lagged a little bit during lockdown and COVID and all those restrictions and stuff […] I didn’t like having conversations with people over Messenger so I stopped talking to people. (Carpet fitter*,* M*,* 30*,* UK)*

DT served as a necessary substitute during lockdowns but quickly lost its appeal once restrictions were lifted. DT was a functional tool, yet it could not provide the depth and emotional richness of in-person connections, leading to, as participants described, a deliberate retreat from digital spaces once physical meetings were possible again. Especially, prolonged dependence and reliance on technology for social connection were associated with feelings of disconnection and the loss of authentic, organic communication.

*I think perhaps during lockdown it [DT] was a way to keep in touch with people*,* and it became even more useful. And I think*,* after lockdown*,* I perhaps sort of stopped using it as much to be honest. […] I would prefer to have face-to-face interaction over digital interactions. (Digital marketing teacher*,* F*,* 28*,* UK)*

#### Digital disengagement

Digital communication in professional and academic domains lacked the informal moments that naturally occur in face-to-face settings. The reduced sense of personal connection, coupled with the option to turn off cameras, contributed to feelings of anonymity and a diffusion of responsibility and in turn limited active participation. This made relationships more transactional and less personal, diminishing the sense of belonging, emotional connection and engagement within teams or classrooms. For many, the absence of casual social interactions fostered loneliness and made it harder to form meaningful connections, especially in new online relationships. In educational settings, students reported a lack of opportunities for fostering group cohesion. Colleagues and classmates were reduced to voices or names on a screen, which lacked the warmth and emotional nuance of in-person encounters that typically strengthen bonds.

*I’d say it’s [video calls for work] mainly positive*,* but it’s obviously it’s kind of impersonal*,* like when I started my job*,* I haven’t met any colleagues for four months*,* so it’s that*,* you’ll lose that as well. You’ll lose the “Ohh*,* let’s go for a drink after work” or “let’s go for something to eat after work” aspect. So socially*,* you could say*,* I guess*,* socially in real life is kind of diminished a little bit. (PhD student in psychology*,* company owner*,* F*,* 35*,* UK)*

*When I summarize it*,* the benefits [of a home office] don’t outweigh the negatives anymore. […] The negatives are in people losing social contact. In our company*,* people are losing that social contact. People have to talk to each other*,* because otherwise it’s tough*,* and we miss a lot of that small talk in the kitchen or just in the toilet or the canteen. People are also losing a lot of skills*,* such as verbal expression. I often see it in online meetings that*,* if it’s like a hybrid meeting*,* the people sitting in that meeting room communicate*,* but the ones connected online*,* 98 per cent*,* are silent. (IT*,* F*,* 50*,* Czechia)*

*I had such an observation about myself that when work moved online because the pandemic started*,* I started working with people I didn’t know before. While talking to these people*,* we didn’t use web cameras*,* so I didn’t really see for months how these people look like*,* apart from some photos that were on the chat. How they look like*,* how they behave. And I definitely noticed that it had a significant impact on the smaller bond I was forming with them – compared to the time when I came to the office every day and worked with other colleagues. (Business analyst*,* F*,* 34*,* Poland)*

*I’ll take the example of work again […]the students for example*,* even us teachers*,* we needed to have this contact with the students and everything so it’s the same in our private circle. The exchanges we have with people and everything. I mean*,* we’re human beings*,* we’re not robots*,* so we need yeah…” (Teacher*,* F*,* 24*,* Switzerland)*

## Discussion

The findings of this study contribute to the literature on digital experiences tied to the COVID-19 pandemic. Unlike previous research, our study specifically focused on examining how peri-pandemic DT use and its impact on the basic psychological needs of autonomy, competence and relatedness are recalled today, i.e. three years following the official end of the pandemic. Our analysis suggests that individuals remember peri-pandemic digitalisation as a force that fundamentally reshaped how they experienced and negotiated their basic psychological needs during the COVID-19 crisis. At the same time, the findings indicate that DTs were not described as inherently beneficial or harmful by participants. Rather, their recalled effects on need satisfaction and frustration were portrayed as shaped by broader social, institutional, and material conditions.

The results of our study refer to the first of Kranzberg’s laws: “Technology is neither good nor bad; nor is it neutral” (Kranzberg, 1986). However, our study moves beyond the rather general statement that DTs have “mixed effects”. Participants’ retrospective accounts allowed us to identify the specific socio-material conditions through which DTs were remembered to have either supported or frustrated psychological well-being during the pandemic. These included time availability, spatial arrangements, infrastructural adequacy, organisational power relations, and caregiving responsibilities. Together, these conditions shaped whether DTs were associated with experiences of enhanced or reduced autonomy, competence, and relatedness. We argue that identifying these mechanisms represents the study’s main theoretical contribution.

Participants frequently described DTs as simultaneously enabling and constraining. On the one hand, digital tools were recalled as facilitating continuity during the crisis, enabling remote work, social connection, participation in important life events, the acquisition of new skills, and the maintenance of social support networks. Many respondents associated DTs with greater flexibility, efficiency, and new opportunities for organising everyday life. On the other hand, participants also described experiences of exhaustion, pressure, permanent connectivity, and blurred boundaries between work and private life. These findings highlight that the highly ambivalent and context-dependent character of human–technology relations manifest itself in retrospective narratives of digital experiences.

This complexity contributes to the ongoing discourse in cyberpsychology that cautions against monocausal and technologically deterministic interpretations of digital harm (Ferguson et al., 2024; Mansfield et al., 2025). A recent paper by Mansfield and colleagues (2025) emphasises the importance of evaluating research on digital harm (especially among youth) stating that monocausal technological determinism should be rejected to conduct high-quality research and provide evidenced-based recommendations for the beneficial use of DTs. It is crucial to investigate the role of DTs fully, presenting both negative and positive aspects and a wider range of contributing factors.

Participants in our study frequently interpreted challenges associated with digitalisation, such as exhaustion, multitasking, digital exclusion, or difficulties balancing care and work both as individual shortcomings and broader institutional, infrastructural, and socio-economic conditions. These accounts suggest that participants understood the rapid shift to digital infrastructures as interacting with pre-existing structural inequalities consistent with previous research (Czymara et al., 2021; Battle-Fisher, 2021; Reichelt et al., 2020). The sudden closure of schools, collapse of support networks, and competing demands of remote work and caregiving disproportionately affected those already marginalized (Černohorská et al., 2023). These findings align with the principles of Self-Determination Theory, which suggests that social environments and opportunities for meaningful agency shape individuals’ capacity to fulfil the need for autonomy (Ryan & Deci, 2017). Similarly, participants’ descriptions of gendered care burdens, organisational power dynamics, and digital exclusion resonate with relational approaches to autonomy that emphasise the extent to which autonomy is embedded in social relationships, institutional arrangements, and unequal power structures (Brown & Browne, 2023). In our material, autonomy was often recalled as constrained precisely where digitalisation interacted with these structural vulnerabilities.

Interestingly, our results suggest that digital engagement was remembered to encompass shared interest, cultural experiences, interaction spaces with new norms, social support practices and dynamics. Participants’ accounts of group chats, online communities, livestreamed events and hospital tablet use point to digitally mediated spaces where belonging, participation and support were actively negotiated during lockdowns. From the perspective of Neo-ecological Theory (Navarro & Tudge, 2022), these digitally mediated spaces can be understood as social-ecological microcontexts that shaped belonging, participation, and support during lockdowns. Our data thus suggest that social enrichment was remembered not simply as an effect of “being online”, but as emerging from the complex dynamics of particular virtual microcontexts, including their norms, affordances and patterns of interaction.

Finally, our findings highlight the importance of how DTs are introduced, organised, and regulated. Sudden and poorly supported forms of digitalisation were often perceived to undermine autonomy and well-being, whereas supportive organisational arrangements were remembered as facilitating adaptation and engagement. From a technology acceptance perspective, the competence frustration described by participants is consistent with the collapse of facilitating conditions reported throughout the pandemic. These conditions are the organisational and infrastructural supports that research has repeatedly identified as crucial for the successful adoption of technology (Venkatesh et al., 2003; Dewi et al., 2023). In our interviews, memories of unclear instructions, faulty platforms, lack of user support and unrealistic performance expectations illustrated how the absence of such facilitating conditions was associated with experiences of overload and reduced self-efficacy. Conversely, collaborative problem-solving, remote work options instead of furlough, and flexible digital arrangements were remembered as supporting competence and engagement. Models such as the Technology Integration Model (Shaw et al., 2018) may be useful for interpreting these patterns and guiding institutional practices in preparation for future crises.

More broadly, participants’ accounts in our study show that memories of DT use were closely intertwined with references to general well-being, reflecting the close relationship between general and digital well-being (Vanden Abeele, 2021). Participants’ recollections of context-dependent enrichment and exhaustion suggest that digital well-being should be approached not only as a subjective experience that can be attained through individual effort but also as a structural and collective issue, requiring equitable access to infrastructure, realistic organisational expectations, and need-supportive digital policies.

## Limitations and future studies

While our study relied on a relatively large, culturally diverse participant sample comprising individuals from six European countries and therefore identified recollections of digital experiences likely to be of relevance in a European context, the study had a few limitations: (1) Our findings were derived from the analysis of a multinational European qualitative dataset. However, cross-cultural comparisons of participant narratives were not possible as individuals were recruited using convenience sampling and were therefore not representative of the population of their respective countries of residence. Thus, our findings are neither indicative of the experiences of the European population as a whole nor of the inhabitants of entire individual countries. (2) In order to ensure that narratives about the pandemic would be unprompted, spontaneous and therefore a reflection of salient aspects in participants’ digital experiences, the interview schedule used did not include pre-determined questions about participants’ experience of DTs during the pandemic but focused on general everyday DT use. Therefore, it is possible that our results do not capture all relevant memories of peri-pandemic digital life that might have been important to interviewees. (3) As our participant sample mainly comprised university-educated individuals, certain difficulties in the relationship with DTs that are more common among individuals from lower socioeconomic backgrounds might only have been captured to a limited extent. (4) We did not analyse differences between how peri-pandemic DT use was remembered and how digital engagement was experienced at the time of the interviews.

To gain a deeper understanding of how salient recollections of DT use during crises might compare to current digital experiences, future studies should consider investigating both recollections of digital life and current digital engagement. This would aid in the identification of the precise conditions under which DTs can maximise need satisfaction both during and after crises, enabling more evidence-based preparation for future health crises, such as the identification of concrete measures to increase digital well-being. In addition, future research would benefit from the elicitation of participant narratives about digital life through direct and targeted questioning and the involvement of a more socioeconomically diverse representative participant sample.

## Conclusions

This study provides novel insights into how digitalisation during the COVID-19 pandemic is remembered retrospectively in a European context three years after the official end of the pandemic. The study specifically examined recalled experiences of peri-pandemic digital life that were relevant to the satisfaction of the basic psychological needs of autonomy, competence and relatedness during the COVID-19 crisis. Participants’ recollections suggested that digitalisation was remembered both as an enabler and as a barrier to need fulfilment, depending on a range of contextual factors.

More specifically, DTs were described as offering the possibility of enhanced temporal and spatial autonomy, especially for individuals with access to time, private space, and sufficient digital infrastructure. However, these benefits were not universally recalled. Those with limited living space, or inadequate equipment frequently reported encountering new burdens and constraints rather than empowerment. Participants’ accounts thus pointed to an interaction between digital tools and broader socio-material conditions. It was not digitalisation alone that was perceived to lead to greater control or flexibility, but its perceived effects were described as conditional upon time availability, spatial arrangements, and cultural understandings of self-determination.

Similarly, while digitalisation was remembered as offering opportunities for enhanced competence, such as improving work efficiency, it was also associated with structural weaknesses by participants. Participants’ testimonies suggest that inadequate digital infrastructure and rapid system changes often contributed to cognitive overload and reduced self-efficacy. Moreover, participants’ sense of social relatedness was described as having been both strengthened and challenged: DTs were reported to facilitate vital new forms of connection and participation during periods of physical isolation but also fell short of replacing the richness of in-person interaction. These experiences indicate that digitalisation was recalled as a double-edged sword with context-dependent effects rather than a straightforward means of improving well-being.

## Supplementary Information

Below is the link to the electronic supplementary material.

Supplementary Material 1 (DOCX 33.7 KB)

## Data Availability

The authors do not have permission to make the data publicly available.
