# Supplementary material for: Connected and confined: memories of digital experiences in relation to autonomy, competence, and relatedness during the COVID-19 pandemic
Source: Curr Psychol. 2026 Sep 18;45(18):1514. doi: 10.1007/s12144-026-09895-9 (PMC13588911; doi:10.1007/s12144-026-09895-9)
Supplement: Supplementary file 1 [file 12144_2026_9895_MOESM1_ESM.docx]

Table 1a. Sample age and gender.

| **Participants** | **Total** | **UK** | **Germany** | **Poland** | **Czechia** | **Switzerland** | **Spain** |
| --- | --- | --- | --- | --- | --- | --- | --- |
| *N* | 148 | 32 | 16 | 26 | 28 | 33 | 13 |
| **Age**  mean (SD) | 42.04 (16.14) | 44.06 (17.88) | 39.06 (13.16) | 43.84 (15.56) | 43.71 (14.65) | 37.79  (17.55) | 45.08 (14.28) |
| **Gender**, n (%) | | | | | | | |
| Women | 84 (56.76) | 17 (53.12) | 7  (43.75) | 17 (68.00) | 13  (46.43) | 18  (56.25) | 10 (76.92) |
| Men | 64 (43.24) | 15 (46.88) | 9  (56.25) | 8  (32.00) | 15  (53.57) | 14  (43.75) | 3 (23.08) |
| Other gender identity | 0 | 0 | 0 | 0 | 0 | 0 | 0 |
| Missing | 0 | 0 | 0 | 0 | 0 | 0 | 0 |

| Table 1b. Participants education | | | | | | | |
| --- | --- | --- | --- | --- | --- | --- | --- |
| **Country** | | **Education (type II)** | | **Frequency** | | **Percent** | |
| Czechia |  | Bachelor or higher |  | 19 |  | 67.857 |  |
|  |  | Below bachelor |  | 9 |  | 32.143 |  |
|  |  | Missing |  | 0 |  | 0 |  |
|  |  | Missing |  | 0 |  | 0 |  |
|  |  | Total |  | 28 |  | 100 |  |
| Germany |  | Bachelor or higher |  | 13 |  | 81.25 |  |
|  |  | Below bachelor |  | 3 |  | 18.75 |  |
|  |  | Missing |  | 0 |  | 0 |  |
|  |  | Missing |  | 0 |  | 0 |  |
|  |  | Total |  | 16 |  | 100 |  |
| Poland |  | Bachelor or higher |  | 23 |  | 88.462 |  |
|  |  | Below bachelor |  | 3 |  | 11.538 |  |
|  |  | Missing |  | 0 |  | 0 |  |
|  |  | Missing |  | 0 |  | 0 |  |
|  |  | Total |  | 26 |  | 100 |  |
| Spain |  | Bachelor or higher |  | 11 |  | 84.615 |  |
|  |  | Below bachelor |  | 2 |  | 15.385 |  |
|  |  | Missing |  | 0 |  | 0 |  |
|  |  | Missing |  | 0 |  | 0 |  |
|  |  | Total |  | 13 |  | 100 |  |
| Switzerland |  | Bachelor or higher |  | 15 |  | 45.455 |  |
|  |  | Below bachelor |  | 15 |  | 45.455 |  |
|  |  | Missing |  | 3 |  | 9.091 |  |
|  |  | Missing |  | 0 |  | 0 |  |
|  |  | Total |  | 33 |  | 100 |  |
| UK |  | Bachelor or higher |  | 20 |  | 62.5 |  |
|  |  | Below bachelor |  | 12 |  | 37.5 |  |
|  |  | Missing |  | 0 |  | 0 |  |
|  |  | Missing |  | 0 |  | 0 |  |
|  |  | Total |  | 32 |  | 100 |  |
|  | | | | | | | |
